# Environmental Contamination during Influenza A Virus (H5N1) Outbreaks, Cambodia, 2006

**DOI:** 10.3201/eid1408.070912

**Published:** 2008-08

**Authors:** Sirenda Vong, Sowath Ly, Sek Mardy, Davun Holl, Philippe Buchy

**Affiliations:** * Institut Pasteur in Cambodia, Phnom Penh, Cambodia; † National Veterinary Research Institute, Phnom Penh

**Keywords:** H5N1, Cambodia, environment, outbreak, survival, natural setting, dispatch

## Abstract

To determine potential risk for bird-to-human transmission during influenza A virus (H5N1) outbreaks among backyard poultry in rural Cambodia, we collected environmental specimens. Viral RNA was detected in 27 (35%) of 77 specimens of mud, pond water, water plants, and soil swabs. Our results underscore the need for regular disinfection of poultry areas.

By June 19, 2007, the current epizootic of influenza A virus (H5N1) had caused 317 human cases in 12 countries, including 7 patients in Cambodia, all of whom died (*1*). Direct contact between infected bird secretions and human respiratory mucosa is thought to play an major role in poultry-to-human transmission (*2*). The role of indirect contact in virus transmission remains poorly understood. A few studies have suggested that some avian influenza viruses can be maintained in water fowl populations by waterborne transmission (*3*). Moreover, experimental studies have shown many types of avian influenza viruses could persist for a few months in cold waters or up to 8 days in feces at 22ºC (*4*). However, results obtained with various subtypes of influenza A virus may not apply to the current H5N1 subtype. Further, data are lacking regarding the survival of subtype H5N1 in natural settings and conditions.As an exploratory step, we have introduced environmental sampling during responses to influenza (H5N1) outbreaks. This report summarizes the results of the environmental investigations conducted in 3 villages with influenza virus (H5N1)–associated outbreaks in Kampong Cham and Prey Veng provinces, Cambodia, February–August 2006.

## The Study

Cambodia is tropical and remains hot (24°–38ºC) all year with a rainy (May–October) and a dry (November–April) season. In response to notification of a confirmed case of influenza subtype H5N1 infection in humans or poultry, we surveyed all households located within a 1-km radius of the outbreak site. We gathered data on proportion of deaths in poulty flocks and on interaction with other species by conducting interviews. We also collected corresponding environmental specimens in some households and their surroundings, selected by proximity to the index household. We swabbed surfaces and collected materials by using 10-mL sterile flasks in the areas where poultry were reported to be free ranging. Swabs were placed in 1.5-mL virus transport medium; all environmental samples were transported at 4°C within 36 hours to Institut Pasteur in Cambodia for subtype H5N1 testing by real-time reverse transcription–PCR (rRT-PCR) after RNA extraction by using a viral RNA kit (QIAamp, QIAGEN, Valencia, CA, USA) and for virus isolation after inoculation onto MDCK cells. From each household’s flock, we collected sick poultry and carcasses for subtype H5N1 virus testing, sampled 10 randomly selected ducks, and bled and swabbed cloacae and tracheas. Swab specimens were tested by hemagglutination after egg inoculation; positive samples were confirmed by rRT-PCR, and serum samples were tested by hemagglutination-inhibition assay with H5 antigens provided by the World Animal Health Reference Laboratory (Weybridge, UK). An influenza (H5N1)–associated household was defined as a household or a village poultry farm where 1) an influenza (H5N1)–infected patient resided, 2) influenza (H5N1) was identified in poultry, or 3) duck serum specimens were positive by hemagglutination-inhibition test for anti–influenza (H5N1) antibodies (*5*). Of note, none of the poultry owners who were interviewed reported having been vaccinated against “bird flu” (data not shown).

We collected a total of 167 environmental samples collected in 43 households; of 77 samples collected in 14 household areas, 27 (35%) were found positive for subtype H5N1 by rRT-PCR. Of these 14, the median positivity rate per household was 50% (range 9%–100%). Viral RNA was frequently detected in poultry feces (50%), soil swab specimens (50%), water plants in households’ ponds (50%), swabs collected from feathers of recently dead poultry (50%), followed by results from mud collection (29%) (Appendix Table 1). The subtype H5N1 genome was similarly identified in moist and dry surfaces (38% vs. 57%, p = 0.41). Viral loads were highest in contaminated mud (mean 94,000 copies). However, no viruses were subsequently isolated from the positive environmental specimens after 5 passages on MDCK cells.

All initially surveyed households owned chickens (5%), ducks (31%), or both (64%), although most poultry flocks were small (median 20, range 1–60 for chickens median 141, range 2–1,600 for ducks). All poultry were free ranging, and mixing between chickens and ducks was common. Deaths had occurred in the previous 3 months among 29 (67%) of the 43 households’ flocks, although the flock mortality rate had a wide range (30%–100%). Of the 14 influenza (H5N1)–associated household areas, 4 had no evidence of influenza (H5N1) infection in poultry flocks, including household 12 in which no poultry died (Appendix Table 2).

No association was found between positive environmental results and flock deaths or subtype H5N1–infected flocks. Of the 29 households at which poultry died, the median interval between the sampling date and death of the last bird was shorter among the 10 households for which environmental samples were positive (median days 0.5 vs. 16, p = 0.005) compared with 19 households with environmental samples with negative results. In addition, viral RNA was found to be detectable in the environment up to 12 days after the end of the flock outbreak. This RNA was present in soil beneath poultry cages with a viral load of 11,000 copies.

## Conclusions

Our findings demonstrate that viral RNA was frequently present on various environmental surfaces or materials in the influenza (H5N1)–associated households and their surroundings. The presence of viral genome in water and feces supports R. Webster’s finding (R. Webster, unpub. data) that the viruses could remain detectable in water and wet feces up to 4–6 days at 37°C (*6*). In addition, using regular techniques, we detected viral RNA in small volumes of unconcentrated water and in pond water plants, which suggests that levels of influenza A virus (H5N1) in these contaminated waters might have been relatively high (*6*). Notably, mud collection and dry soil swabbing have been efficient in detecting viral RNA in a contaminated environment. Nonetheless, the presence of RNA does not necessarily imply that the virus is alive or that transmission can occur; in addition, we were unable to isolate the virus by culture. This lack of culture growth may be related to a number of factors, including the fact that viruses could be short lived, whereas the decay of subtype H5N1 RNA may have been sufficiently slow to enable detection by rRT-PCR. Also, a live virus adsorbed on soil microparticles may have prevented viral binding onto MDCK cells, or these inoculated cell lines may have been damaged by bacteria or fungi present in the environmental specimens (*7*).

We used the interval between the last dead bird and the sample collection dates as a potential reflection of the survival of the virus in a natural setting. However, this interval may be subject to some limitations. First, we were not able to prove that infectious viruses were recovered after this interval. Second, these viruses could have been shed by duck survivors a long time after the end of the outbreak. Finally, interpretations were difficult because our analyses were limited by the modest number of flocks studied. Notably, however, an interval of 12 days was reported in 1 household, although none of the remaining birds was infected or had markers of influenza (H5N1) infection; this suggests that the virus was shed by the last dead birds infected and detected 12 days later.

Bird-to-human transmission is believed to occur largely through direct contact between infected bird secretions and human respiratory mucosa by inhalation of infectious droplets or transfer with contaminated hands to the upper respiratory tract through the nose, mouth,or conjunctival mucosa; subtype H5N1 has been understood to replicate primarily in the human respiratory tract (*7*–*9*). However, additional evidence suggests that influenza virus (H5N1) also replicates in the gastrointestinal tract, which indicates that ingestion of contaminated food (e.g., drinking duck blood) or water is not a negligible source of transmission (*6*,*10*–*12*). Most rural Cambodian households possess small ponds (≈10–20 m^2^), which serve as water reservoirs for backyard animals and gardens. Ducks gather and deposit large amounts of feces in these ponds, while at the same time children commonly bath and play in them. Taken together, widespread dissemination of the virus in a subtype H5N1–infected household and high interaction between humans and poultry, the birds’ environment may be particularly worrisome (*13*). On the other hand, current strains of subtype H5N1 may not yet easily be transmitted from poultry to humans; however, this transmission could increase as the virus continues to circulate and evolve (*3*,*14*). In addition to illustrating the need for good poultry-handling practices, our results underscore the importance of the following for preventing disease transmission: general basic hygiene, fencing domestic birds, and regular environmental disinfection of poultry places (*3*,*15*).

## Supplementary Material

Appendix Table 1 Influenza A virus (H5N1) detection in environmental specimens collected in 3 villages of Kampong Cham and Prey Veng provinces, Cambodia, February–August 2006

Appendix Table 2 Poultry death characteristics and positive environmental specimens by collection site in 3 villages of Kampong Cham and Prey Veng provinces, Cambodia, February–August 2006*
