# Supplementary material for: Environmental Contamination during Influenza A Virus (H5N1) Outbreaks, Cambodia, 2006
Source: Emerg Infect Dis. 2008 Aug;14(8):1303–5. doi: 10.3201/eid1408.070912 (PMC2600401; doi:10.3201/eid1408.070912)
Supplement: Appendix Table 1 — Influenza A virus (H5N1) detection in environmental specimens collected in 3 villages of Kampong Cham and Prey Veng provinces, Cambodia, February–August 2006 [file 07-0912_appT1-s1.pdf]

Appendix Table 1. Influenza A virus (H5N1) detection in environmental specimens collected in 3 villages of Kampong Cham and Prey Veng provinces, Cambodia, February–August 2006

Specimen source	Wet or dry specimen	Out or in household yards	Environmental specimens		Viral load, copies		
			Total	No. (%) positive*	Mean	Maximum	Minimum
Wet feces	Wet	In	4	2 (50)	2,300	2,300	2,300
Water							
Pond water	Wet	In	1	0			
Poultry ranging place in wetlands	Wet	Out	3	1 (33)	16,000	16,000	
Pond water plants	Wet	In	2	1 (50)	16,000	16,000	
Mud							
Poultry ranging place in wetlands	Wet	Out	7	1 (14)	510,000	510,000	
Mud beneath animal cages (poultry, pigs)	Wet	In	4	2 (50)	880	1,000	760
Mud by the lake shore	Wet	Out	1	1 (100)	45,000	45,000	
Mud from ponds	Wet	In	19	4 (21)	5,150	8,700	1,600
Mud in household's yard	Wet	In	3	1 (33)	1,700	1,700	
Dry soil in household yard	Dry	In	1	1 (100)	1,800	1,800	
Soil swab							
Beneath houses on stilts	Dry	In	7	3 (43)	4,350	7,300	1,400
Poultry places	Dry	In	7	5 (71)	10,500	20,000	1,000
Pig places	Dry	In	4	0			
Poultry ranging place in wetlands	Wet	Out	4	2 (50)	7,700	8,000	7,400
Surface swab							
Feathers of dead poultry	Dry	In	6	3 (50)	48,000	85,000	11,000
Chicken and pig cages	Dry	In	2	0			
House wall	Dry	In	1	0			
Rice straw stock	Dry	In	1	0			
Total			77	27			

*Specimens tested positive by reverse transcription–PCR; of these, no virus was isolated.

Äœ¼Íx orÇø Ï¡ÕÕ‹F½HÝÕ’åâßk□™XÈ□□À#ª□á°□¡,^"□,È/□ □üö4O„žf÷oh<{sóãÏÕéå‡□ÿóéóÿÁÆû—
7ßÞè‰¹□ü□yûÙ›ÓOŸXFc□□ùéÉ
ËD¦y&Z□î^í`ÔÄ^□'E&cìß'on¾¸ýÉùŽMÌ□yûÍ™OJ/[ÏcëmlÝÇÖWç;2qÉ

»ýë™²‰*%nOç;1Í,)yûäl/É□ÂèíÓÈôel½Nì^□™g•³“øã”~<□Í¤9ÓìöWéÇ·±ç‹ôc&þc¤'E‰çog;ŒÊh□V®ÍøÉ™□IÌ2ÿí»È’□Áû³˜¨`:S÷á|''A
„^HüËmèùË³œ

□FÞŠÈó»ø[□€¿œ□G&æ¾pU□‰üüLÌ$µR·Ï¼□”0□èBè`9%ôöÝùÿžüú†h6□¥ìLyrogÆÛÈ□*2½]□–£—Æ÷b°□7

□1Ú*O,Aã□ÛQND2V”øà'□□   Þ1¦ÂÝv¢3spü•40©q´-□ãÉ_□
›8#º¦;ðd"OÑŒLPºœÌ¤¬YÔþP□Áûkn□+1□ï@Ù¼ä□/³□óÓC™‰h□¦Çb□¢¦wQý‹ôc□§ßx´ÑFi«¨p³ÓMzW¸í/K□ü3ï.ÊÊyå‘Er□ds/z¢R(’
³<□□ßÆÖ}le□ÿ□ÌMóõyìùC□Öt…ÿ›Xž□+’-—%-ßÄWNá□+Þ»$1›DÉºìÞ|□-;□->[ä×îfÞ1Éì□ÓÓ□™(‡;ú³□<
-Ã½¡<□îwÛ±□G_*«<ÃíØüï□□R|ù2ÞÛ□Œ>½$âØÛaþ{$¦Xö±0grT

CúP4□Æžs¡%qâZ

7’lžÝ °□”0Ž¿HSà*□

–□ zXMè5æ¤9YAÁĐz–™æAPˆ}□□óì®Þ¥□f22O74¤□Ä
8q)ü

{sc–zòÿ[
r□f□RHO2w*Ž[rår□x□□¹□□ÉëÑE?+¡Õ□Õ=KòÖ¯rPO®×<2Â^I§□ùÅ“›?Þ□ÓW7f¦vFY°□þ/sÿ□Í‰âß÷Ïo^d□ô¢□+vÒtÑ‰pÂ—=¬?ªm□Ô^@¤p

¦-{p¾'ƒ‹$C□eìÚÁr;Œ.È`FïÉºè"ìð¿'e□>s¾-
ÃöÐi@□□@>□Ÿ□*&¾ÌÎÿTÈÎ□f^¦/L†□Çu1yOéó‡õäŽ¸é[□h□□F□¹7·ýEØTs2´Çé)×6E…«þóùŽò‰Ùl,Ë--²@-¥□ËÀà
!#ß□ÈyÐÎ□"±k#%x-□was=ú^›˜pä¸Ù:nTs7□□ãÆìâDr□·ßŸíd¡nØR¬{È□-uÜ□™FÊ'óü□ði&Ìäãõª□¼],…b¢â□,±d□□î_fÜ□…åZºŠûL!"±□Öo
oŒš□bÔ|z"ý¯Eœ×7d-
lÕ¶Ý^ß¼ºùóém"WoÞX&äÄWXÿÇ£`aUº□(ÝZÛBôÌI6"•□□RjEÑ†□é4hãþ4MIààdŽ®ü»˜□'«¦â"ÄE±»‡ÿq¾ã"¶¿r·ð¥6[äF^dÌ«ió¡äŽåånÊÅÒL
üÑÙ^hÛ^p;sr'□óËú.□□bÜüŠÿk#‰ŸSóªm»õÌ¯Í¨Ám„§ëdbÄ□□6Üá½ü-□VÉH¾ÒÈ—Rv˜í□«¶A□v

’Æ□□□\aLÉMyjo«½□ÄÞÖTƒÊîu-È~L«BÍ\□o¼ß.{R]¦Ê9Ü□ÜÔâúm×Ô6□¥Í™×·ßhVÈ©¸É2&ìÁX5s‹=²ÌM2q)c‘1qN
=x5/‹=²¼Ìf)□□†Í;2

ãe□GœÁ^±\¦f—IŠ

IŠK¯²ådV“L=□iUø±iñàM!³Â°¿?'=□□fÒ›éU□ÝwÕSÅ§F□ðý™õÌ□¥ü*arSb□□Ø<±ÆÄ□□ì°□□–□†m□š5§^ž□S□ç®‰–
□•,¢¥ênª#ØÐÆÚ`□Ô‰ÙN–<¨c'Â□B

¶¹Ïa□□«ö x□ÖŒ□7^n□nhÄÈÏòòføqÎZ□'□4ØÀÓZ□□□+œÉÇµI‰©Ç$9§ÅÒ#™$©U±Nrlâ"¶Ë□Øc£&□=ª□ O®W#<¹^Š□rµ

□äZ□□ÈÛ°□å□vÁ]□¤H®…Q W+□H®•–<¹^³□d]

Œ@®Fg$×B3□«q9□–
!(ÛÑŸu5*?Í¢r□€S□Ã*Ús□Ð`ìjB□†}□□□*□□dã□"vÁ ü6‰ä6]3¾„¡f#'†Eä□æv¡□Á□Ú□àÔ"Ö²=□ÌÁÚG@s□,□Å êGB2Jl.¼lÆë·WœŠž$"□□sÜ
‘=2DÚbž!9ýÌÝ,!ÎRGöÈPç¶äô³¸äÞÖMsÝì,Û#C□Û’ÓÏ’]p;dØà¶‹ÙÌò□□□Ù#C□Û’e6jóJ·G†□n®Í□·C†□nÃh™ûÈì³C`Å.□ÁÎ9–]ÑŠà¶Þr'«t
¾!Ëá¿?ÛéªmhÌöÆ®‡□0□8‘m □

Ãrx□×žÄ□Ã‘,–%t¤ñÈ‘òx²—Ç#GG"□□. a–bnÓáÖ·□ÙÑ·

›cý□Û€ÙÌ&□Ëö üFsÆã7□nÅï;ÎŒ□□®□ì□ò~ßßÒNCvN'ó(¨èRm□□û□ùiÁ…Ó¦o¶–½qŽÀIL5Å×□[ÙJ]ì³¯Ù□ŠVG0–□R<¬³Ðy¹□\¨joMRi□□
øF7×–€c□À ¸v€□□FSå¸¼™Ñ¥ßÎö‰7",r$´a{H€□□h□□yÝ€ìÔ□X¤³+ê□¨¯R8`3□X□mÄ'¥VíAX…æŒÇ*□lL¬□“¸ê•¥åñ|dy

Ú„õŽÆ%;2□æ2âOežõ□Ñ5-%Ñ
g¹sKc

',,Üï

Ó7q
ˆ¾□åu”(□d&² !□us…0Ù
PÀ0

Q\;@□Ã‘

%td`È‘0‘îa"rt`"pô`â±Z*ê«a¢&   □uÊÙ¸□«ö LDsÆc"
þD˜□Ú:0□□>□&¢¶

L□Ž5&B™Â:ÄV™Â‘7Ê□H®•)□y£L□äZ™Â“·uo”)□y£L□äZ™Â“ëe
$×Ê□Ž¼Q¦@r-LáÉõ2□’ke
O®—)□ü□e
ëö«□_¸[Lu¶@°oAw/J«ŽB¥ç¸²NÉeÚt,6`£`bÕ–„“ÞØG€I/·¯JÉ…Úr□GÞp□$×Üß‘7Ü–
É5÷÷ämÝ□îïÈ□î□äšû{rÝý‘\s□GÞp□$×Üß“ëî□äšû{rÝý‘ü□îoˆ[íöT)ÿ]5Ù¬šhÃÅõU□¸□=U□à□¶(@qí‹□`□V5Aq□U□äH+□µ·B@ŽŽ□□pô¬□□
□_±B@}•□,`©Â
m^v’□U{PäCsÆ‡>□lÔ¤p\EÌ¬I!Û–•Œƒ^A¥õŽž□ ì□ k™Oj©2‹™ºoéV)%□–Î€¾œó□j)é ¬ÒÑþô[±Òòª„¿¥RÊòQ□w ‘ˆË'Œ□æ¦‡
2œ/?@˜€¸□–Òó°¥ƒ½Kª□1Ï□□□„CÌÓD‘Êƒgßd×□%½Ìl‹□áÑbo□□Î³s□Juë□¢À1(`□qÍ□□□

T',„ö*Ràh?□□8Úc□r|²□yA_%FHÂbŒ€6Æ□-Wí11"˜3<F `1
,œ|q«#Œên@□â,<6>Q□8²#xd4Ì□□Ígð□ÁFy¼Œ/lÚsY¼êÔ/íŠ»Ñ‹É\]4\□KŠ+…uàXE˜S=□÷žôõÅéæe^ŸAmé□ øðYË□gn8ˆœ;□o□þ:Yíàk{□JÕ
½¬ö<Ýwo?Há»w ¯íÎ©Ât®'=/=^Ý

ÂN–`□ø5©8□m€\%Åª=□~□!ã±×IÍÊ÷<□‹□Ž€VU{íÞ÷Î
÷□mX{ÕÞußÊ½
GSšñòE©Š’
3é©Ûâîeéñþ¾ê□%Þ~âÎÙ´â□ç□□v ®□ï<Ã‘d□%t$›È¡dLX□Ú˜

- Z ¸=ñ

•yîÉw□0å¦ä□Ø□}•ÜSÉTŸ€6□žædÕ–□~`Î#àŸ□œ=□R„□R@Ÿvx□□íO„□Ž,–
Õ%«Ô!Ð□□íù(0ä□…□“dyÜ¨õS![VÙ®L–OT×µè5ýÕ□dWeòÞ§Î□š□Ìÿy&vjP£
o;r°ë□¢3Ý´m‡ƒD¤j^□□Ç0TGqí¨

GP□%t :r´Î
□□I,p|²ÃyA_□Æ5O%□h□t□¢WíA0ŽæŒ‡q□œÁx□›

□&ªè€qäØ++Œºª□Æ□¡å□ÞÏã›ºv6üŠ/yÉ□uQÌÝØ•“ê±!·P$8Ž®L9ç"’û□o¹□ÀAå□7E~–ß`‘¿C¦!aGQŠµ¾ç□9Ž@+Jè€Väè(

G□´□Ç?£D€šKU□COñgh#ª□µj□BX´e<Â,à=„ÕG°

Ut ,rì!ì□«Ú□□□Ê§úŒ½Í□Çz□yãX□’kÇz□yãX□’kÇz<y[÷Æ±–GÞ8ÖƒäÚ±–O®ëAríX□#o□ëAríX□'×□õ
¹v¬Ç“ëÇz□<þX□µ£Ok¯qÛ□Â…÷³•ÎÐ□Ù□

¼%dn=ä

ý¯;□èÞ=□ð□Û-Cé¬Äª=□O½±□□§^nß‘@ç›[Øa6□□"¹Š□fóH ’«Øa6□□"¹Š□fóH ’«Øa6□□"¹Š□fóH ’«Øa6□□"¹Š□fóH ’-
□;ÜkâxµhòÓˆ#×-□i«ž□Þu½s„£Tà.bÙCq™ñ:ž9yÈ˜□’l□/"õÔ˜‹□Ã□□Eu$øÀq$ÁG □ >r´Ñ
□□ >päµ□¶“à§□ù5µ□ÔW®□Ø(£R$J□3R"èª=(□¡9ãC□□Î2{^Ê¡ÓË~®(□£ŠŽÌ-9²Ì^m[uEf□:Ú3{`Ø-
□óãFõ”À¥p+ÞOxÀ»ò¼dñ¥s]»□p%„□„ÍÖk□´(ª□h□ã□Ð¢„

EŽ

EŽ

□Ž□hÅ

ÐªC{□¨¯□´Dð□´Ð□p¥Ô¬Úƒ€□Í□□´(¸ø´dñ\±8□$ –ãÄ□2d‡.è¶y‡

] ¶Žs□À±‹¾□¬êA_å^‰°□¾ë³Àë□¹□S±□Þ□kÞ>âŠQ□ÓÀ3

AT□Ô□¨žá□ž,€

8□†ŽSÃÀÐ□¦ž¡ãÝ?ÈP~õ□¥,Õ? □□Èf±j□□C°f<□z¹W¼ø§ðdÃà

Õ¡õ1êèÈÐ

»x□úOËox-^□"□ÂMð_|•zù□ÄÕ;)`)±0ÕŠ+Àqåf
Ó2¹¨Ž§8)—dÕ–ä¢`í#¸(□îÚN±WÌ6¶S<¹¾□□È•í□O®o§□re;□ÈÛºëÛ)ž\ßN   äÊv
□«Û)□\ÙNñäúvJ W¶S€\ÝN   äÊv
□«Û)□<~;…ÙÈ êgP‹"ÜÙ;)½°¬|8ô]Ä¢

µv>ßú´□ø□SÕòã±i□e•N³¶u!Ž—ÛNi\□"Ç^Ei□Õ¾*EŽ□ËÒ ¡}]□8R¾(wòÅÀÑž/"GÏ~Ê¡|1è«ä‹vu□ƒ□´!□ÙYµj□
FÁœáÁ(□.>U5<ICmíe¾À□•ù'QÃŸ-
ÚÚË|È!Ýy□¾.ó□/ø□mÖ5□×²

–È–¡}ú¼mBólñže‘_—JzÅ“°¥□Ä

r_=pÕ› F„HÚš¶"Ç<GQx

□Gð□%tà9r´¿ä2ptà9pt”□□G¥Ö(XüL□¶□…%á«ö DFsÆ#2□–Smüetð–cé–
/ª}□ŸP¡tï‹‰ÎVÒµJDŽ!î†¢:Ü□8Ž¸□Jèp7äh?o–8:Ü□8zÜ□9*î&IZ□C□]Ì□U{□»¡9ãÝ□□□q·Ï£»•£hîeÛ□ÁJ5ýäŽåJÎÃÅBKå_2ÿ°Åc`Úº Gª□
ßD…□CÜ□Eu¸+p□qW”Ðá®ÈÑá®ÈÑá®ÀÑã®ÈQsW“Ö+Ð□□UR¯ÚƒÜ□Í□ï® ∅
wu·÷–¿
Œ□Ž□□JùkS|³òí4p□n}Ë,²TÊ B+«?;£¢šf±□Ùñˆ>rp÷½µ‹ý—

•œóâ□Y©øÖplÃ$Ó\®Ú£&□˜ó□□Î
î«Ö*¹õ:lOÞ¨Ö"¹V–uä□j–’kÕZOÞÖ½Q–UrëuØ□\«Özr½Z‹äZµ□±ªV–Er–ZëÉõj–
’kÕZO®Wk‘| Z++^Æõú#»?r□_ÏŠI·1Ä'"èì□ÓbbdvÅXl<³8glä—ø□o‹1Œù—²Ì□…–lìù64>$□;± Ÿ„ED·Ñ<IÛJ

üK□©²□Ï%ñÞ□mšb4·B□øMúíÝR™Í□£}šé$/ûí»ÔóyAø—é·ÎÄFL.sóßÛld¦f□ÉÄïÐ~š□Â{/…èÜ®HŒº-VríbÍ-Ž.□2*x-
Ä%K²‹Ë†¦tíï□ù-□□"ì□âÔÎ□-
Íþ-Tþ□æ□óU□;□M”ÕÊmFêÚÚŽ‰¸ý¯d|¼ x;^„_JwþU²âyáþÅß>+hˆƒò®pï¾+N,x/>□”eö}Ÿ]ÜÓb×Ë□ÍÈñÚÝšVO†*žÄd□;š}-
ˆ□□Õ□õ5W□_,ÍPß‘ë_qõäúW\□\ýŠ+’k_q□ò¶îúW\□\ýŠ+’k_q□rõ+®H®}ÅÕ“ë_q□äÊW\□\ýŠ+’k_q□rõ+®□|õW\ 9¹ìþ□ôíZÊ,P□‘Ìü□
å¾endstream
endobj
6 0 obj
5129
endobj
4 0 obj
<</Type/Page/MediaBox [0 0 612 792]
/Rotate 90/Parent 3 0 R
/Resources<</ProcSet[/PDF /Text]
/ExtGState 12 0 R
/Font 13 0 R
>>
/Contents 5 0 R
>>
endobj
3 0 obj
<< /Type /Pages /Kids [
4 0 R
] /Count 1
/Rotate 90>>
endobj
1 0 obj
<</Type /Catalog /Pages 3 0 R
>>
endobj
7 0 obj
<</Type/ExtGState
/OPM 1>>endobj
12 0 obj
<</R7
7 0 R>>
endobj
13 0 obj
<</R11
11 0 R/R9
9 0 R>>
endobj
14 0 obj

<</Subtype/Type1C/Filter/FlateDecode/Length 15 0 R>>stream
xœeOÍJÃ@□ÞM,‚¦þ]µ0 HK[H‹—ÐcŠ§□!ÔzÞš5]ÜÝ„üTò

[illegible]/žK¼ö([illegible],[illegible]àÅg[illegible]ú[illegible]›£‰ –„ù†™ïg`024„16'LÐ¤ç…,Èz?–[illegible]qy¢•M½‰Æ+½±4õ¥i<©÷#õv¨^÷ÕË[illegible]Ò1~þøtÂ([illegible]Y0O¡uå]·;[illegible]î–
Ó·m[illegible]fù[illegible][illegible]#š°@ÂY5,([illegible]#Ae:[illegible]§rsÎn ày4O€ø>õëØ”pz[illegible][illegible]Œ³(
[illegible]ÐrÚ0°¬~¯jƒ!¸™ qØ[illegible]&o™di

Dúp)h@@□ŸÖ□F,¥q

ç“¿i—‰Y-À÷§à†6ŒÁ£AÆIü_Aa¨€4Œmõ°WÕ}QN
5)¶Ö;›Ýõ£inVf¡/<gÛ
endstream
endobj
15 0 obj
286
endobj
16 0 obj
<</Subtype/Type1C/Filter/FlateDecode/Length 17 0 R>>stream
xœuWyTS×ö¾1äæª

JzAA“(*ƒ□29ƒŠÌ□¢ XPD□□$□□d°`-ÚA□Öj□j- 8 -□*Š

ÊàHU ¢□4¨□4*>mõ½}Ó□Öû□
íëê¿•¬Ü!gï»÷·¿óí}□”Ñ□J □

HPf'd%ÇÅòWŽœµ€□3„□+ü□§þá¡Ï□□¥□‡ž› c!26*-c~Þœó□ Uf□;,□
□¹;

.HKÏËHNLÊ’Û…‡.µŸ<Ùñï;.3gÎ”¯Êûë¹OBfrbª|□9ÉNP¦¥«□R³fË□□ÕJerœ<Q™—ž”)□□□O˜çÍ"b•   )r¿derzzZ¶Ün□½ÜuêT—
)äÇua²jÕ°LyXlj¦\!□MH\§ŒÍøÇMŠ¢¼æç¥Æ□{¯O‹_¸ =!Ägõ"ßŒD¿Ì¤Ð¬ä°€uK□³Sr”±□çªV-µ³w□;:M□èâêæî1múŒ}3gÛRÔx*Š
¡|('Ê†ZF-¢|)gj□µ˜ò£¦R□)□Ê…šD…Q□”-µ„
¤Ü¨p*ˆr§ì©□Êƒr -R
ê□*˜ò¦¦SŽT$µ□Z@Í †RÃ¨á”€2¡Ì¨‘□M™S□ê#Š¥,(Kj□5šJ£¬¨1T□5—Ô‡2¢fQŸS□□□□·àž€□â9äÐ□□Â)Â¯…/Œ¼□6□U□ýWd+J□ýAKé-ô-ñ

ñZñ.f,ãÏlbŠ˜
Œz¨ÉÐyC#†¾□f>Ì□XÁ°ŽáÎÃ“†□

□`,4¦□□Œg□□kbbâb²Ç”5□4=jÆšÍ7«7ûuÄü□™#vÁ)S½3*,(57µPÀ¥B□û□§‰ÞÓx□^%ÂÆ4>Ð¯□ý‹î€4□|§fq>□ÆðTdÊ}□S5ú□ˆÍ(®ˆÅ□□ŽØŠs□
¹Òàk(N™±aunA□□ý□*Ø‘·“™'>´éÐWGÐqT°çÈ□Ç

<òC%□èMG™rõ¨^[ÜžThÞÖáõ□¾é²”œo#

%?□Šž@¥XÒþŸ¦¶gw*ã□Rüß.N.î

jšä□©òŽ-JÎkÅ$Œ¬

Î±Cpª[È¥À;ö«Ë□□Î)M¬U”z#□□sÆFØ□{öÊa<Œì|□í•M§
¦¯ŒõAŒó’□0□,□ÔÏÛ.®òÞ+3ÕßGEzŸBÁ□–·[+ä’, –□‘□´z□fÅ8©ß

+õf"Ìö«´□Ð□g□□□ž□vj _cÞ©ƒNÝB□¥„ë´□□ÝŠ.-
>[ÉH>”U□^¹j…´qõNeŒ„»sêLÃ}+TŸS□p:áLä□¾$ÐZZ‡ï²□□□â+¨tËÑÜc9ß«P□Š/P®ÍÉÈMÝ²”,
ÀÏXØ 9â*trã‘LâùHÖþ”D+”ôYÊ°¬u™ª□+□C□Aíœi»€
!0□¬½¡ŠÆw

Æ¢yœW;V‹á□Î„T¯~□¶²n¨ï□ÂQ®ŒÅ¶®ã±□öÑŽ□{°íý□ó Ðý=ž,ÛîÅ¾lòÂ□Øl±§³Sh□HÀüªZ'ã□¨—□q
5x□šë†+¤xùÜf½Š□□Kè¤ú°□|jc\±□Ï–Ij±o7□À¸{uÇoWI%ù~÷Åx=□Ëö^□□Gò□›,žíæ¼ð1˜,éÍÇÏ¥¦úÕƒåx¨å¤Z¡ÞŠøu3èhÃHN'šÄ—
@Ks&□-è□M-fi¸>□ F'Ô;pˆM□wg5vGöhŽ2<$pA¢□ÂÃ□6.ŸÐêÓ´ø^ú+□~èíë#□ÎL¦7G~žœŸª
      Nö$áNt□□, D
b□w½1/í¤ìxÆAå¾pf _ÎAM¸ÚÀsõr·¥äç□œÊ>□côÅðÚÌ□Ä€u□□€dÛ

|†e0□¯‘Î4□×ß°1V`Ÿñäà„□m^□□@À{rp’ñUÒp·4,–□7F'ì±€Ó4(@□RÈ□ll□R□,ÃSi];5ËÝ,yb˜ðÐ□/ÄŠ¹öx,Ì□N‘íïß□Öí□m—
□ö□Ê°kè3…?Vìþ□m;,m□çîÜ¸}=b¼¢cæÉ\}ýÛ

Ë»¸åÝâAÕ:¯□=□Ás□ôô   ÉSo±□G_EU‡Îž¹xîp5ºËÀØY□x,□7ô«t4gm□{ BÜS¿löì^e®²ÁÍû□□
4,^□□>†§,7Nc□□□ÜT□aG¿Š+Ô□¼iSxFÂôÖ'Ê·'Ê[JÊ[y‰*å%ê¨□=8\{®"'´-;|t×µíÌSqÁŽ-
Û7¢p´*e™'#)□Edª-§¶ÁH~Ï□'¥ÝpžxyÌM%næŠ%}·b?)□ºÆ£]0ƒ½<Š}/DÊÎÆ4-mDwÐ¥□Õw˜t1òÙ´2;u□reÞ'(□%ïÍú1÷àæï¿<ÅL£÷Øu,□3¤Fw
□ž®<Wsð

3†8"Žqn0Û[?[â'ásÝ¦.Ø¼Mê–Â8
øýòÔá¿#©Ë5†–¯ÿ‰ku7l"úÄ™²Û~÷¿†M!Mç(fVÆÃ•L{– 8%ŒŽŠZã½"áÀ'lé†ƒ_ü²œq£wb"–Å0–
ìŒQOn¿SÇ\°9*›}Øÿ‡Ô#¨|tÍ…S–wËR—ìþÙîÁÜBA[iÂ6²ÝûU]¤ðàøñMù…Ò'õ{3Ðjf°hç¯Èú8J
'Åb—x%6×é÷Îê³"|€…@Du
ÝU–ç"“pÛ,ÐŠïb

¨Fƒ¨˜N*Ùrlkß60Î–›sšH©æÜÏ—»¬À|N+¶‘âž□¾È□ˆÜ®#Š|rsqÎÑì□kP

ã□□o'%¼Íjç□Z□O iw“"zÀ„□x,□ÿ¯□9□Q¦°]X¸ãÛ□Ò6ñÆ¯7lû□1‰Ÿï-—□~&6…

’gN!□□
î□[□b;ÃPÔEëUFÓÇ?!JæLvê;µy□6P□o´þ¤›tp:n?»□lì»ð□,¼»ÝL<Êµ:ìu†

□—‰$=/2+W‡Z¡øì8å°Ôœè□‹Ð□□~8©2ý§ÏÎl¯"EØ–p ¦4®ÎÿI"□Q7úµ°°¬öì©Û¨□õ†Ý±;Ž?®□%ép+I9Ñduÿví□`nG8n□`ž"5…,®—
Ü0"□5„%ø©N¯rÂ–†áÐâÔ¯z   OyzgµGòy™ßî,□¤□ärÙ□\Ñ

\'-TÉÃ|Ý□cÏ5K9ñ

ƒ□Øývøo□µÇ[k¥’\oñ□□õ´F□ß□□þGoÍÂf□¡u&lé·&

äõ□ï□o_
!,0b
y ÏË□□□Pa□Ñ\‘h
□í

žXÎy’SP□ŠD□–áq¾Fp–Ç7%žN+éÇ□À□—‹~§ñ□xM”>P$§q□Î□□äË»å.[€Ó€[□ƒã□2ÔL¤m□ŽrrâLƒ#ñþ’~Ç9ÿÛà,□('–
Ë□!8’gLïW‘"□8Ú|Û¹©□æ•Ýp`□ŽŸjYIÕÖM¡/–S?=xL□7Å:ÿ:ÌÎU¬‹O’f¦□®Ú°”yBïþ¥¢T□˜□•i‘²ub””□¸□□ËÏû*eÃÂ

åräÏHr□ï„üûN}qÃué®ˆc™□è□:°ãønÒ"ÀŸEi›×gd%+W}□…˜ „Sõ□å'z

È´û□Üyâ□3 š|¸Ü□
ØEæ;;□¯0ôã5\¿È–†ƒdÒûß’NRŽyð□
ÃÉg¯a˜□†Ð¸žó□:¨□˜
ÿk□‰8□a€NüwAª „□ØÅ—Ä□‡bs□□9Ò°□ïÅÁ¸PôŠ†Ñ□□-8ZÔG□Ì[□Î~O □ÿZJjùá□×U□XòøaãÍ–[g□□¤¸Ÿ¿Á_Þ,‹□ä/¹±â—
áWì}c³□EI•M±%þÈ□E¯]¡`$µ÷Åÿ□M□°à
éÕù□□’òà®XR»ð\}j»5H{I§ò□/□□X°à“ôEñ2(□Ã$\Äê□Ûtð?Ûô>ƒàèó– ˜àgCc+C□žÅõ□üð4r:–œÚÐ□B ü□†éÜk˜cxMxòL–
Y(h□,„□$□[l©ÀA□» ìÚŒƒÞbK¦‘v□›(˜ƒÀ□□[-xja<ó§□,x,Ôá$ö-X6C□□□□®
□²□Kf –Å6µD□È°ƒÝ¢°§□&f¤ÁófîäQßb£q□¹‹€Rù¹Ö%6ën9].óí□êdîõq ß,□ íÌùbÛŠžBA÷□a÷{ÖæFž□ùmÈY¾•I□·□©z&□c1¿□ß
°ß
!Œ¬°y?@òvN–□\è…ò^!œÐG²□($uuTtp-#")áÍ□°□□ÁACšN□H×½Xþ‹tuÝ¢□□ˆñ4zU=™ð#:zŠ½Óò7□□□Õ¯^□€MrÛ
°–¨□V´í,□”¶BL+Øñ¿BX¦ŸÏ:yyM™ÒáõR×Ññ¢oÞÃÉ2_£«U+B—D¯X□□}©±éRU£ŒszÉ□d¤□whNFŠtÀ`ÉÛ9ñv/Ô□/^ÿm□>`WÕÔÈÛ□ô =y
,ýZ!ìçv°†□Z}äb±□
ròñ˜õŒ,'□êæ*;□ÜÌ^!÷M7k□Jï¹öSy÷å·□£þÕp¹□ÈëõÌÆØŠØŠ¥?□ □□□□’‘°1fë□FK□S³«t□qñ…KÇê□óèFèÜ°5Ë□‰2ç¥ØnúÊ€ÍxêhN9□¬□Š–
@~‡yµ□

ôúõ’7
X
jv-
]¾]$ùÐW□3ÍuI„óäå5¿□!#=6tïêC)eþmI}¤ÏNzÙ□V½IuÓJIË»sìçº_-ÀtV;¶Æ6s=ñä¯¥Zzû…□%ûŽ□ž½XÒˆ□uuô¼È<Uü§²ÌÏ•_□oc□p□ûNNA
xD x□^,öÒ,×‡ÎñÜÊ 1~`□□9‰áJ'‹3hÈ€fÑ`ÐÝjpî□p³ 4é„

†!t„ÁBô˜>_{ú□yÒ«□Aã'.
žìµüì£l□÷þÅ{ÓŠÖž
¿§|Dâ¶yû–□@îô□□‰ŒÛ Z%;□Q"¨ù□□›ê®¢gjÁÅ–¸@¼□IDb=0s[1…<‘oB¬"Æ/‡ì□□áá?Î–TT„ÞRªùi¼ï–È`”‹

K|—¤/M□m‡ùM¯ß¡[èBü÷þ

Ùá–ìÓúEŽ®!A³f□·<×Þ¸Ù=È□î□Iä□ŸDª;'¡±'þÂ□ÛEà□æ%}□rz`)¸Còà;□üt2Õæå½A½°□üXJ~ç¬¹¡¬¤¯ýbIÙ]+□°«‰□ŽtóÄÂÀ’Ä{+¤’ßgÅÇ–
ò´Â□o\@

ò7½ÀvÆ]ŸyVŠ□ØG

ÔÄø□FÅÌŸuñnKíÅû2\g$ù]{#lÆô□0□□□«]]7š□Þ□`Ë=□w□n³†æ□\óŸh…ªáV‡ ¢—□«–¦±‡0óÐ□(2#þZq¾¹ªµ°□□KÆü¼‡±ÍñWƒOñ/>Ö

¶X†Gõ:ÁGwkK/É¶áùa“'¡`´òlA
qkXÍ–g÷…Á–Ó"®¿ê½qû±ì/éàf¼ÀBH!•áåa<nýÿ„$Lï[(¨y)¬!èb¡ø8¶£ƒ•„bÓ¬b®ˆïÚŠbZ=¬k¸ú[cã§{ŒM(êÿí§lµ
endstream
endobj
17 0 obj
4233
endobj
11 0 obj
<</BaseFont/SNDABN+Times-Roman/FontDescriptor 10 0 R/Type/Font
/FirstChar 32/LastChar 32/Widths[
250]
/Encoding/WinAnsiEncoding/Subtype/Type1>>
endobj
9 0 obj
<</BaseFont/KGVIXH+Helvetica/FontDescriptor 8 0 R/Type/Font
/FirstChar 32/LastChar 150/Widths[
278 0 0 0 0 889 0 191 333 333 389 0 278 0 278 0
556 556 556 556 556 556 556 556 556 556 0 278 0 0 0 0
0 667 667 722 722 667 611 0 722 278 0 667 0 833 722 778
667 0 722 667 611 0 667 944 0 0 0 0 0 0 0 0
0 556 556 500 556 556 278 556 556 222 0 500 222 833 556 556
556 0 333 500 278 556 500 722 500 500 500 0 0 0 0 0
0 0 0 0 0 0 0 0 0 0 0 0 0 0 0 0
0 0 0 0 0 0 556]
/Encoding/WinAnsiEncoding/Subtype/Type1>>
endobj
10 0 obj
<</Type/FontDescriptor/FontName/SNDABN+Times-Roman/FontBBox[0 0 1000 1000]/Flags 5
/Ascent 0
/CapHeight 0
/Descent 0
/ItalicAngle 0
/StemV 0
/AvgWidth 250
/MaxWidth 250
/MissingWidth 250
/CharSet(/space)/FontFile3 14 0 R>>
endobj
8 0 obj
<</Type/FontDescriptor/FontName/KGVIXH+Helvetica/FontBBox[-5 -218 929 741]/Flags 4
/Ascent 741
/CapHeight 741

```
/Descent -218
/ItalicAngle 0
/StemV 139
/MissingWidth 278
/CharSet(/two/A/y/n/c/three/M/B/z/o/d/four/N/C/p/e/five/O/D/f/six/P/E/r/g/seven/F/s/h/eight/R/endash/t/i/ni
ne/S/H/u/T/I/v/k/semicolon/w/l/a/V/K/x/quotesingle/m/b/W/parenleft/parenright/asterisk/space/comma/period/z
ero/percent/one)/FontFile3 16 0 R>>
endobj
2 0 obj
<</Producer(GPL Ghostscript 8.15)
/CreationDate(D:20081204100321)
/ModDate(D:20081204100321)
/Title(Microsoft Word - Document1)
/Creator(PScript5.dll Version 5.2.2)
/Author(heu5)>>endobj
xref
0 18
0000000000 65535 f
0000005464 00000 n
0000011703 00000 n
0000005395 00000 n
0000005234 00000 n
0000000015 00000 n
0000005214 00000 n
0000005512 00000 n
0000011270 00000 n
0000010516 00000 n
0000011031 00000 n
0000010356 00000 n
0000005553 00000 n
0000005583 00000 n
0000005624 00000 n
0000005996 00000 n
0000006016 00000 n
0000010335 00000 n
trailer
<< /Size 18 /Root 1 0 R /Info 2 0 R
/ID [(□[-Đ‰6Ä–□²ÕÁ+b“Ø)(□[-Đ‰6Ä–□²ÕÁ+b“Ø)]
>>
startxref
11898
%%EOF
```
