# Supplementary material for: Environmental Contamination during Influenza A Virus (H5N1) Outbreaks, Cambodia, 2006
Source: Emerg Infect Dis. 2008 Aug;14(8):1303–5. doi: 10.3201/eid1408.070912 (PMC2600401; doi:10.3201/eid1408.070912)
Supplement: Appendix Table 2 — Poultry death characteristics and positive environmental specimens by collection site in 3 villages of Kampong Cham and Prey Veng provinces, Cambodia, February–August 2006* [file 07-0912_appT2-s2.pdf]

Appendix Table 2. Poultry death characteristics and positive environmental specimens by collection site in 3 villages of Kampong Cham and Prey Veng provinces, Cambodia, February–August 2006*

Outbreak investigation date	Village	Sampling site, household	Initial duck flock size	Initial chicken flock size	Duck death rate, %	Chicken death rate, %	Testing in poultry		Time interval, d†	Environmental specimens	
							Serology (HI test)	Real-time RT-PCR		Total no. samples	No. positive samples (%)
Feb 11	1	1	320	7	0	0	Positive	Negative	NA	4	1 (25)
Feb 11	1	2	220	0	91	NA	Positive	Negative	1	4	1 (25)
Feb 11	1	3	100	0	83	NA	Negative	Negative	12	2	1 (50)
Apr 7	2	Subtype H5N1 patient's household	2	30	100	43	No ducks to be tested	Negative‡	0	23	2 (*9*)
Aug 13	3	5	14	0	71	NA	Negative	Negative	0	2	1 (50)
Aug 13	3	6	25	8	76	50	Positive	Negative	2	1	1 (100)
Aug 13	3	7	50	8	70	50	Positive	Negative	0	3	2 (67)
Aug 13	3	8	20	30	90	60	Positive	Negative	6	2	1 (50)
Aug 13	3	9	6	20	0	0	Positive	Negative	NA	4	1 (25)
Aug 13	3	10	5	50	0	80	Negative	Negative	0	2	1 (50)
Aug 13	3	11	10	25	80	84	Positive	Negative	3	2	1 (50)
Aug 13	3	12	4	2	0	0	Negative	Negative	NA	2	1 (50)
Aug 13	3	13	1,600	0	80	NA	Positive	Negative	0	19	10 (53)
Aug 13	3	14	Unknown	Unknown	Unknown	Unknown	Positive	Negative	Unknown	7	3 (43)

*HI, hemagglutination inhibition; RT-PCR, reverse transcription–PCR; NA, not applicable.
†Interval between the sampling date and date of the last poultry deaths.
‡Tested on 1 sick chicken.

œ~ødó›Sh□ —
ëÍé#h$□çÂ»Í^@…à˜□Álœ`!ÀÏÓ''_n?Úí□SAØíó□fÎ÷©ËšzZS□5õx·□L[□Ôöß;©˜tÎl7»½aÜ(g·§;˜R□BÉíYmt^S7-yê'pîpsY3YËÜì□óZyµ
ý¬ŽüY-ø°¦□ðW-õ‹□aÒ(¿ý~□Ð¹à□îð¢¶nã9«)$æz'□³ÞuÃ}XE^§6□R-uí¹e=Üí-3ÂÙ€e_.›
œ_ÖNP“Ç;¡˜VÊb‘/©□æe°ZåõLÝ\ìþyú§□á%3*□MN/€□¥□1>³!Õl87áelRÈiD±□Êý¶V],r¬Ù2Û^¾¥¤g4Dðh□Oë:<□Š- □\'q*FC;›S$
Ï¬’\ÀÌ*,Ï□□&A□à,°1SçV)²s$□Mü¼ÖÄ\œ†□úyF□□%ÉÁÑkA!‡†4XÕD□□EÚMô —□5zJ•+*“\ë›
-šg[¸+bŸ.è-…·ù>j™□ä?'□âƒ‹¬Hxh¼-ÏëfyÖo›”°ª)ÔáÇ;Ë,p□ëu-îD3i□□h

g„è¬‡T‡Ógm¬m'\Ö&
}□Û|ÑÚ\-0□+½C“žm¸”‡W+Ñ"□æ½œhÑd#*^NóoKt¯□□-µ□çh5JéEÍz<£IÊìz□Û□VÏÇAï□‘

F¶Ù□&u-ùï›©j6¯™ªŒ…„¡¿®ygDiY□□ü§ZÚ,lk{US¯□ƒ)s@ÚCN¤lMÝÛ
fa1cã8_Í96óAÆéÚ<Ûh˜%ç\‹n□¡þ'zÎ÷ô˜údÍ¹□™í¦×5Õv<Ò\□□□W□("□Ìn□×èý”dèÈvÌöÁ\}—âB²¹é(Ã=Àt¤š—
hK´‘□,GßõË´|¿;=ù□Ž}□G□Û□ Úlž _□ÚÜœ□0ßä¯Pùæäúä-
›§ost□i”pr„qÇs#Ìúô_ydŸŸˆÍc□NéÐ™ôSÅÿó°‘åç‹Ë“G¨’ï*)²R™ÂT□çŸô{WIp°I«b€,¢□&□B†°U†B24)Ãß*Ã#□Š’!åm2¤¼m□ÒÝ*Ãa□š□□>
Â-2□°}}□ÊÞ*Ã!□□’¡o•¡±

r.FÜ†‡□□□/E“q□Û□9Z□Ý□²’V(d□□ž§lÍ%ak¾Æ‡Ié’AËfì/'§÷¾Ä¾BsÙfgð”G›□ÔüŠèýUõ¾PÅg„“XÇ□ÇtAJê=-□šªØ+□À:{“ýád¯¾¨&□u{C&
‡§DÔ□Ò,8í7¡vð·ÚÁÙÜ¥°Â O□õ4G°tJx†/□NƒZ□□šß¯øV□-sÜéî°rMxdä9¢ ¦š ¹\Ì—□ÐàÜnZŸŸ´á¶Ù"□h

agk²yèLpA

¿™É□Õëù□□Ua”À^<j½ßÌ^¹&”°ÜèÔðŸù®©ðhË¤Rn   OœÔ□ž±o|
<è□Ý"

¨99+Ê¡îàsÌh“□ƒ3øbŸèds4ø€]Âs□àûm=úc"T©_SÞÍAÑšXñú`□Ú9‘□„÷)áNþj·×Ì□□ôD□Í¸sxž3ÿpÎ’nÊäŠ□^ûÛâ
À□*□Žˆƒ□□æ´□ké,Ö·Í3Eñ®>ÂCÒ±ivÂí□¥SÁ

xÚxV_mk□□¬ëð
éŠv“%â8¨ü«]‘ê¦Ù:Æ…Âº óAí–□òI_b_□Øé‹Uoa1qÉ¸²□–[ÿ9"k£žl}¡ÐBãçÔfÚ³púæ\□˜•“Í‰□Yšëõ!□□à¶□WÊ»□!}Æ9ÑfÁˆv–!!¤C
–RðC?*□™ê□¢&šÖ3jL□'°Wÿ}œ,□□□œ9ám¿õ□ÉÖcRË□ ®□ý¤€`U@ñ"*~Z□¸MŽ‘□Qþúò Ó;ìã°o¡*ŽÂ□¦„3úÎ'ß‡YaÓKXP□ì¤—
Dˆ{V©xþ□ÈÁ□Ü&‡9€w]ÒÉ—□□TWŸ†:wáT+□□ Ñ©ö~áU□¯–®”

99¥†–;lS±ÃVjôŽÕ‹1CWªÖÀ®å]¹R`D½î]©[,uèä–j¦@g½³5ó9¯¤cONÎ2•–ºžáªDÅ=ìpk
–=PéR¦=Ë„–XbUŒ?¶üì]WF§´L´ûÛ¦lÁ¼3JÝ–né([
8CÅ&û£2‰¬N¸–«–ÆKÇ@Œ™e(DfZÖrSS
{›ö
lL¹–|*5V"O¹ÆZØ)ÕÇœJñ(à”‹‡Ñ¦R<
ñäâaœ©,L©xa*Å«}cK¥xXJÅã¨R.–j¨\<Œåb·Úz¬ sñzßã8T.–¡2Ÿ†¨R¼ºbc¥[Š¡qÁ=
öj\Ii\y¨ÆíõhH|jf?
zÐ›'®ÒNËØ÷û¨…è©ÉzUj¸dÒÞ©îM¾€„µ”åcp>´ì§¼Ã¿©½iEÑSåIãk¦zÒZŒËùªÎUÒ>€22~ªTÇ¡ŒôÑ#CD¨ÔH¥™ˆoÀÆ^'ø
P?/Lø~ª€ØC]Lj¡ñÒ¯@]Ç¨âiº×)RƒB¼–Ät>_•HÆSÀ@0_JÅD0àY˜ZüiÍÃ¬–
¡\8€ÊM–Ïx~>fÈ,”JÅõPÍ_ïìu|ï•žaê&€x§AdÇ·AÁ©„©â\÷ªÞÁ¬A[¹ß«fäý›ZƒÚ¶.«¹åþUñc¨s×w~…ŽE!Hò¦
ÚxÏvðô‰Ã¾¥r

Ž! Ÿ”´ä<ú“ÚÈ>□ëÜ…oéÀ’étš‘Ë/¶o#Ø
Ð□□m\ü‘

□çãm²ˆ¥5~Íî±õÅ‹ï□ž□žElîZ!ªí□„jä□ž□5Â$Öˆ
7Q,ƒô^_"Õˆâ□<□¢Â
äbJˆbKC\$Œ¾†s†pj¡9}Óœ:Æû6{é¦Û□ë□NH³Û□¼2ÑÞÄ□»k¨□Ü¶¨î–g.&–mó”ßèˆöÞZÛr@ë)U

homd‡ÎqÞ1ùÓðQya)^4uo(^øu^Ø®□ƒýå□â_g□´‡}□ ½ÇGwI1£#□æ°Éõ–Ä□□1:Šäx¾`,«þ

_òÈSØ□8ã(Î

□ø@Îðÿ–Î□ÚDuœ)Údd—&ÒØZîÖ´I»hrTfxŠ□Ã@À<FÛ□Û□UQ($@rÄþb9b)ŽÜ¥Å9 $0□ÛŽA□|Ì

"EXã□I□pÈé›)ír:Õqù^dþÍçƒyªåÓ□¼¤□¯–r□

æ÷àd.6ñß‹+Ô#ð$□+6†ë□†û|QÓµšp-i5ïâ□□T6~Æ□>~‰Ïgó°□”G@y□”G@y

””ÒN-RúfJ7 Jš□Ê# ô*P-□¥

?
PR«
”]□JJp¥ê´!]§ít›vJO@9□”C@9□”□□•ziŒ2c R¿ÇfTÖ□□(Z
/ s□2‡ s□2‡!□7§B–Ò¥…*Þqª“Ó4dNc Ö s□2~□È”Ñé¦D„Œ¯p+½È«Ó

¦M[š6m‰6¡²®j«”žZ8□”□□•ß□–¢–R¿Çæ–□¢nÂU ,□Ê" □□Êa ´h@¥ti¡□£ô
£R/‡□åÞ□P–3î3PÇ9ŽÌ!×ˆ›□qS#nêŽ›□qS#njÄM½ÂM}07õ»à¦□žÉ□ù□Žh
¼□Å5¢¸F□×□Å□hv&¥o¦tº¢RêL×U□¼1ÅµQ+xw□çÇ1ÈÆ□¦□xã□Ð□²è04□š□□m □□ØÎ□ƒ
3□2ÓLsI“□¥^□□+v&õÛÎ0Ç¡¨Uíºw¼;□àEAoþø¡□Éß□ä)ƒ□□□□9□V3¾øæ—¿□yº□¶ñŽòÿ£Œ□G□□□□XÒ¢□□–Ïº#Ö,î□ôáÈ□!□paŠ□´â.Èý
ˆ□¨£ºåM?ü–3F@□HRzEã6`□@·Šþ£ååwútPñÞê{¨D

»Ñw<uò

ÕH×□@□>QéýÖÍ,>HE
C„õ¶«%3söK¿□D~Am$ ¨‰r°Ý¤é>_$□"4‹×□–□™„ä•□ój?‰z÷§˜Îd(Ïá¤–·□¯—EÚ•

ôî§e¶Uÿn'□1Âèé–e–(¥×0‹"1.å[K´NÄ□¼K‰ÞÆ;Îw'Q©xIQÜÁþH□%ÀÌ□SyöÎ¶Št@□.æ¸$9s\fï1æ¸L□ h

“mr□,îmýr#5be#E·ÄÆK¢È–ñÅ–%†´¿<z/NÂs§â•©=gÎ□eÔÛOâÃ□$ÿþN,ñ5iHiè*Þ52L   –Ç–
D|□Âaßª øGSö1j□¼□ôKqMÄ□I t%tñzÊ{5õ□VÃ   §â£Ý*älTÏ«H©ZïéToCÔCòêàQñ9‘×u□Çìâ££R¯vöaC¥öû□□y°ÛÃéW□–¶{à□—
□›¸'4‹ÏdÅöchïXð00Ü

¡^□/jëË6—ow°–”□Â·¥x‘ŠE¼k¶,¬âÊ_

v½ž¶UëãŒÀ□É{˜ÞýH□°µµAØ='×l□"dý4e}□/□2 ãÇ

□§□S€Éé□□À|ŠJ?ÊÀ
Ø±÷‰…©SA´yEð»Rîù"qÓ&‰æ‹ð¨mÏçœ‡yÖu`¥×jý¦•Øç^B\□Ú Ûv|Ú□–ÃEK×">ÃËÃ□h87msU<Î[□—„Üï□!/Û–_Ùq□ÌkBr–õ’@
)□[°F[ùª□¨Ê¾XS□h(ËµFr/□C~c±•ä□□„E□#Eš¶£□¼•o§©Ò□□qFìÆW□ÕŸ□T□M□ûªé—½ôÑPo¿ß□]a;‘³¯s=#□sM

nÉô2I`°(—
â~œÚMÚ□o•,Ë□ë1â+kEÛ„:HAð±ö€LÐÃ6‡¯□ak#TLY□ºõe#Û@□¤ø©Tnù`J;□×M5Æ□□Jñè±S.->v*Å£□G¹xøØ©□□žÎ¤âñc§R¼Ú÷ø±S)-=vJÅãÇ
N¹xøà(□□□åâác§T<~ì”‹×û-?vÊÅÃÇN™OÃÇN¥xuÅÆ□□JñÏ□ì$Ô&†—□§U□ß□wÇÕ-¾'ý_é7y³endstream
endobj
6 0 obj
3839
endobj
4 0 obj
<</Type/Page/MediaBox [0 0 612 792]
/Rotate 90/Parent 3 0 R
/Resources<</ProcSet[/PDF /Text]
/ExtGState 14 0 R
/Font 15 0 R
>>
/Contents 5 0 R
>>
endobj
3 0 obj
<< /Type /Pages /Kids [
4 0 R
] /Count 1
/Rotate 90>>
endobj
1 0 obj
<</Type /Catalog /Pages 3 0 R
>>
endobj
7 0 obj
<</Type/ExtGState
/OPM 1>>endobj
14 0 obj
<</R7
7 0 R>>
endobj
15 0 obj
<</R11
11 0 R/R13
13 0 R/R9
9 0 R>>
endobj
16 0 obj
<</Subtype/Type1C/Filter/FlateDecode/Length 17 0 R>>stream

xœcd`ab`dd□òHÍ)K-ÉLNÔôõOÊÉ,,M□‰jý□fü!ÃôC-¹»äÇëŸ‘¬²

>sÞðvó0wó°Ìû!/ô½[ð{□ÿ÷V□□fFÆŠ)
□ó
*‹2Ó3J□4BƒÂ5µµu□"†---
I•0□□—ÔâÌô<□5 £,5'¿ 75¯ÄZÁ□¨:''3Y!=§² £X!1%%5□¤-,1'5[Á-3'³ ¿LAÃYSÁÈÀÀP□H□ùeæ&•□+□'æ□+ø(□¥¦—æ$□)x-
$□□B‘c```´d`□b`bddéÿ¾ŠïÇ□’□ßƒn|?}CèÞã¸Çß÷>□□þrÿ»™hWCwcw+‡ö□ß¯'Ž,Ü¾_îÌÁõ§—□YòbÎ—î□Ý»*÷ä®É\-
1Ï·Û°Û15Ò…CøM¢□©w€”â□□ï

ÛvÍX¸ZnÆ”ÓV.Iþíáj¨ÖÍ!üÅ;cÓùï…?²DÏï‹ðP´0Q“~ã’¸íò·;OÞÈó•,ú±`þwßù>‹Ønr=æ¾9‡‡%Q¤¹
endstream
endobj
17 0 obj
402
endobj
18 0 obj
<</Subtype/Type1C/Filter/FlateDecode/Length 19 0 R>>stream
xœeOÍJÃ@ÞM,,¦þ]µ0 HK[H‹—ÐcŠ§!ÔzÞš5]ÜÝ„üTò

□/žK¼ö(□,□àÅg□ú□›£‰ –„ù†™ïg`024„16'LÐ¤ç…,Èz?–□qy¢•M½‰Æ+½±4õ¥i<©÷#õv¨^÷ÕË□Ò1~þøtÂ(□Y0O¡uå]·;□î–
Ó·m□fù□□#š°@ÂY5,(□#Ae:□§rsÎn ày4O€ø>õëØ”pz□□Œ³(
□ÐrÚ0°¬~¯jƒ!¸™ qØ□&o™di

Dúp)h@@□ŸÖ□F,¥q

ç□“¿i—‰Y-À÷§à†6ŒÁ£AÆIü_A□a¨€4Œ□mõ°WÕ}QN
5)¶Ö;›Ýõ£inVf□¡/<gÛ
endstream
endobj
19 0 obj
286
endobj
20 0 obj
<</Subtype/Type1C/Filter/FlateDecode/Length 21 0 R>>stream
xœuWyX□×□Ÿ1df”–’Ž(Ø$Š
(²» □Ê

□¢ XQD□□„□□d±`Ýª•qé,J–□□□´‹¸ È¢,®T□**Qƒš€□Å§Oí;“wÃ÷Þ□èk¿þñ¾ä›õžsÏò;¿s†$Œ†□$IŽ□LHÍIÈNŽ‹□îìyk’;ŒÿH´□¥é_ëóÅ–
□Š□ÏL9□□gbT>Öb□□?k$\0‡¼□□□IæíÜï“ž‘Ÿ™œ˜”–·□□[b7uªý_OœÝÝÝå«òÿ÷Fî›□•œ˜&Ÿ„/r□RÓ3” iÙsä>xujjrœ<15?#)K–□–
Ÿ□/ˆEÆ¦&¤Èý“S“32Òsä¶>vr□''çiøà² Y¹jm–<<6–K®□‡%$®M□ÍüÛC, ¼æç§Å–K□_à“‘□ê»z¡_f¢□VRXvrxàÚÅA9)□¹©±‘–
ç)WÙÚM‘ÛOspšèìâê6}ÆÌY{Ý·n›3™ Æ□QD(áK,#□□~„#1□XDø□NÄD"€p&&□áD 1™XL□□®D□□L¸□vD$1□˜B,!□Ä

â□"„ð&f□öÄRb□áC

'F□Æ□I˜□f„91’ □
BB|H°Ä(Â’□MŒ!Ò +"ƒø”□K$□ópŠ□#b6±‘h"MI□2—¼7Œ□-8¬Y4NT
ª□½7r7Š5*□»‰?□·P"*“ú‘ú•^Bo¤ëé□ô□FÂ□0Û˜Z¦}xòð□Ã›†Ãˆ°□ÇG<1¶4ö5

0N1.4Þe¬5Yi²Åä¹i’é□S0K2+6—˜O4/6¯3□ûÁ„□¼á„™Þ‘+ƒ(□ïTJòi□ÎÞCéâw□*Ö+ÅÈ„B%□Jñ?¨.H□Ã·*□□P`□OÄfü—
(M–ÿ□ËŒæËX4Ñ`□¬x{±
□~†ò”YëWç□&qc>ç
wæïfæÑ□6□Øv^;ÊU□úþÈÁý‡¾¯†B½Ùh3¾‘+ã□u&•Ztty>†]Ý–
’³□X¡ä'CÙc¨¦%□ÿjéxz«:N!Eÿéæå´&¸e’ÇR¥w´TrVKc3²»xû.ò„FÄ§À□vÛÅ□?åV&Ö+*½9□□sDFÈ□yôÊa<Œ|p□>Ü#›I□Î\□ëË1Ž‹ïÁ□`Ù¤zÖ
q~•÷–□ÖÅuòf□$Š□0X{C□…n□LÄóxÏN¤¢áWÞ□ûÝ8´áI□4jDp˜?É¢É.ã‘/òÕŽ□;˜Üû–æA□Û;4U¶Ã“íkñD£□ù"□G‡°.□€Åe•N&(Q–
.ã□*ð,µ8¢□KØí□~³^ÉÎB□TRcø1?lýX□D¢92I=òÓ □ÆÝi8z³F*)ð¿K£u|,Û{y

□)¼6]4ÇÕqÁ#0□³ë□žIÍô«¹2½o)y_ËKµ"½□ÖëjÐQ†‘¼N<i@©åµ□ojÐŠßSxi¶šïW“u:‘~
Ï±iàæ¨Bnœ□775"4È'Ñ†C#8dR5¡Ý·eÑ□Œ□□øs¯_–,□f*µyéÆä,4ehH²□6w¢#0□

¡Z aÜÕæüôã²£™ûS÷F0ƒþòST8ËMB-/j,%?5¡4ö1□¡ÎGÔgµq

X÷   ØÛNð

Ã□Ì7,%$-'ù□'-¹ ë□ìók³ÑHd¼`¶“ë¢‡`

æW–j□CÙ□□màÖEž}*â÷Àz–{øE]Áéä§s.Ùa³&MÃ□˜‡æ=□□“Á¤»□¨2Œ□lÿå‰□\$·¢"ý|îñ–
Ç‹.1;ÛØoú¯¶>æ□ukÀôíÜö¢í23ýÁ¡øœÒñûu"~Œ>ŸE h<r@y(□ð□Vk–
□m¸*{p»□Ä□□3ð9’Á4´Fên□.ÿD&H□|Çã“□r´Á¥□□□ïðÉA&dIÍßP“=:~¬NÔ3
~¦@□b□B.ä #□¢□□ú™Ò□X³ü□˜GÃ„ûSÐ□¤ð²C□dfp□□N@□Xw’Ún□ìÅ□uSS¿”þpê›]\ÑAi;□·{ÃŽu□ã□□3OæâDaXÞÍ/×ÐCõ~V□Åjò™

zúEx×□,äS—¹š□§□9□æ`-w›□□fw¡
RÔ4 ÔQ¼µ□□C$ÝÓ¸lÎœÈe.2A‰□v©¡PMöêDð1<aùqjÃ8(ä□Ô†□□J¾Tmð¦Ìà)6Ó[‹3ßŽ3o)©j□Š»R(îÃ4wï`ý™JFÒ~äàá¯¯ì`žÐ…;·ìØ
ÀEp«R-y0’ª□¸À□QZ□Œ□j

+©ÔÀY¬å□ï„ÕxÑ’þ□±ŸT□Z£1Î^AžÓËýÎ–•□Žiù´™»Å]8V{‹É 9ßM+sÒÖ¦®Ìÿ„Kà’÷dÿ□·□ów[O03¨bÛ®…`Î©¸Û‡□®>S·ÿ□□æ

V–Œ□£¼□¶·Ñ
Y¢‘□^®N‹□‹·EÕƒ□□8~¿þÍux£Fo°ërµ¡P^×Ÿ´T«□M˜™,y3¶èmÀ□dŠ□i6W1Å½:->PÊTyW
Ës¸„1QQk¼W$”□Ê‘®ßÿÅþ–UŒ+µ□™¶–,□peŒ~|ó□*æœÍaÙœƒ□ß§□âªÆÔ□;ÑvûdÚâ□Ò?□ô

x•’□Ý˜@E□¸Ü□”Ý˜<□ê<ztSA©´bÝžLn53D¡ZEóÄù+²?Ž’Âqz□,à,□v«-t:@½³û-%ïa□Üfa
Õ]□ç,“ðEÁÜŠocJ”c@\N%Ul9²½¿□LòNÎý™‘¼WŸùéb·□XÌmG6RÔ3ˆ□¹□ì†µô%îøæòÜÃ9%k¸□fzL¼-□ã6»“ŸÒF>Á ý□'q:L˜…&ø□üf□S‘'•7KK
w~uLÚAoør}Ñg□“¸qO•

ĐSÚ

2±Ÿ¹¥□RJÞÆ²S°ì,CY7¥W□Í□ÿ□3™#®Ô7*‹:m□

```
^i□t-’.^Çïc‹ÀÆ®□ÍÅ‘w³uG£]jÃ_fÊ h™XÒó<«zu˜□□Ÿ□—°6-7zÃBn.□q0©:ãÇÏ□ÙQƒ“°#°$¦2®!àq"ˆ8□÷[iíÉúÓ'nr-\oø-
Û£èãúÑ’.×Š”c-VwoÖ¿□æf¤ýŽ!□□ RWJv÷ñ#0qÔa” ':½Ò□µ□Œ¡Ía@Ù□O□xgw.□ü²¸Ù□»q□ÈãsFñe³P□-©‘‡û¹□Åži•òô,ƒ-
ív3üŸ]õGÛë¥’<oz□‡zJMÂw□…ÿÒ[³°Ymhw‡-□Ö□□□?□=àuŸ□"1"¦á□□\
```

□‘„S£ š/□O£□–Á□Éy□|
C™ø□K„8_Áq–'4%□N+©G□Á□U‰ßRh□¼ÄL$–S(□eŠ□»ˆ^ZJ>Ñ□:[□e

Å´□o(□–□Sr8Išxl]□×ÌðÅ

ø9²ýÝ□æƒ□

ëƒIR¾x□ƒ,YüÅQà0h–□Á~□–'&R“□ör|áH□=¶®□zÃ;þnp□□ÂAð¯í±□ìñ~3□”□□8œ□~□¼S—Eµ□J□Ãùc=+©Ù¾i□·Õ:í³ýGdp□Ö□4
ÖK±6>Iš•±Q¹}   ó˜úæ×S•*Ž¹W□¾T¶–æ’r
,6£□□ùÛRÖ/ÈL]Î□0’<û[¡¿ßj,o°*ý:òHV□w€+Ùyô□Üb €åÒ7¯ËÌNN]õY□Ç□'œhl®:ÖS"Óîûa÷±□f□t□sù±£àk<YÙRh…a□–á□Ä“)Ø□g¬?—
<Àéœ□ß²`Œ□{

#Ä0ŒB□¼/4@ÃàDöƒXÌ‹1,tô_ –□
□|ák!¥□(

Y@˜Øž□´□… Rñ

Æ@4X¢hq?®•»□N]□!Cø[Jê…±Kàe5-yt¿ùzÛ□_□□¥h@x Ü^?□$ÜòÑ}□—ìübs□FIS[b+□8?.úÓ□
FR□—þûhÓÔ□—p¯/h□¤¤{·iIý,3□i□Ö íÅ□Î□<§¿GRŸO2□ÆË ’†I¨ŒÕ□µù□¿·yxª_ZJv>□Á□¬k2²T `

9sÈ¥□□¿F-L3å
6Q0—□7

\ëÁC
ã™?ÄÀç‘H‡’Ø×`Ù
Á□8sà¢€àÉ`É,¦´È¦–³□žy□k□òpEX

÷yAÌ□oõ□2□w*o!□J□—†ÄVÝ□‡‹'ýz€8žwu\ÂW`„‘x□ÿÑäS=¥¤æ½HóŽµ¹-Osþës—
og□èŽ_jžÊÀ□ó×SØr‡„*¸É¢M>ºiºDß‘šw"□Ç26ï□ÑÛÉ«Tä¹^¨ê□Á1ýR6ˆ
M[□□□’mÏa'Ñæ‡H□ö0E□»Q!H×>_þ«tuÃÂc□□ãaô¢v*N|tô4;‡å¯ □"k_¼□™ýÛ4¯□¢;Ê:`EÇîR²²□bÚÁV8Š`™~>ëàé9mZ—gŸ®«ëyÿ¼ûSe~F—
kV„–Ž^±8<úBsË…šf□ïÐÇ□d□¤·(^†Ñ[bº□ä□□¹çª®ç/ÿ’‹□”«ii□ä□›“–□XÀ>–□öñ;YÃN–~é":□‘¹□hì:F!
†ÓðÕ]$ïÞ+âwiXÃpªøÊ□Uš‹¯›Fÿ£éb'æÝ«YÍ±§bO–ù!□sæ□SâC3□6Äl÷a´Ô®º¯+÷•—Ÿ»p¤‘c–^
ó
_³L‘(s\,lg®

ÜŒœÆð©ƒ□UAù=(è²¨ÕBI¯□¯¥„‡OAÅî¥ªv^%ïû□bf¸,Žtœ°¼îí□2Ü|Ãö¬>□r2 #©-
7àI}□°êMj˜Q‰{á-#?5üf□f³;‘5²ñò@S¿”j©□çvWì=Tzú|E3Ç¨j£ç-ÍWÆ□&ËÚ˜°-¤^□Œ□Ø=à□8□□q(-,'‹<µàùþÁx~e0□î□ÂÄ

4\zÀ¢L
2¡U<d´F□Ž□’ŸƒC“□á`□FE□F‰Qgë□>€wzq–xüÄ…!S=—Ÿ~˜ƒíÞ·hOzÙ§g"î¤>ÄvÛ¼~□S@îð□□]□·^¹Jv

¢ÄP÷G<®«°ËžªÈó=p

kßŠ«□□0^í^à<8¿„XEŒ□.®   –CÆ?xU+N…ÝHU   czÿk□Áhg□’ø–ÎX’ Û□ó[^¾ánpçâ¿
`péNgŸ4.´w  □ží–ÒöL{íºf□□ü+ìÈ□Á‰47^B!□ô…?²□D…Lu□r{`   ¸AòÐÇ□üx□²T□U½Á½P„□–’·¼5?œ•ôwž¯8yÛ
Dn*Ls#]=□(¨"ñÎ
©äíìø¸…–VhÔ+g□ƒüU/º□â®ºŸ–"□öbÁ{u1þAQ1óçG□¿ÝV□þ®

5□IÞj¯…Ïš□□î<=ôrw÷µ-Á□
¡Nù+¸N□->|ëÑ
SÁ□.òT¯□¬F˜Á-@Ì}□ ððøÛ©³-5í¥□

X<ÿçß□m□¿□rBø"²ž2□ÉÐè^□øðv}Eó□Y□š>u□□Â–<]ØÆÀ‡ü□V}#ÀÑmAÈô□‘W_ô^»ùH0€Ÿ¥†PŒ”Ï´øƒ†/bQ$Ä¹¢8□á

□†>–ß□‘,U'Ôpíÿ/D×†NñZˆ‡H5~uM□ûƒªøYïH□ö^□)8ï□ùŒGíÿ□¸Âõ~¥d]Ÿ¨

Ç

```
‰è£ÈJ|†:
Vb□ÑfÙå|™0,(Ê)Õˆ ncÕW&&OŠML    â¿Q¶Kª
endstream
endobj
21 0 obj
4140
endobj
11 0 obj
<</BaseFont/MMJRJM+Helvetica-Oblique/FontDescriptor 10 0 R/Type/Font
/FirstChar 57/LastChar 57/Widths[ 556]
/Encoding/WinAnsiEncoding/Subtype/Type1>>
endobj
13 0 obj
<</BaseFont/SNDABN+Times-Roman/FontDescriptor 12 0 R/Type/Font
/FirstChar 32/LastChar 32/Widths[
250]
/Encoding/WinAnsiEncoding/Subtype/Type1>>
endobj
9 0 obj
<</BaseFont/BCAJUP+Helvetica/FontDescriptor 8 0 R/Type/Font
/FirstChar 32/LastChar 150/Widths[
278 0 0 0 0 889 0 191 333 333 389 0 278 333 278 0
556 556 556 556 556 556 556 556 556 556 0 278 0 0 0 0
0 667 0 722 722 667 611 0 722 278 0 667 0 0 722 778
667 0 722 667 611 722 667 0 0 0 0 0 0 0 0 0
0 556 556 500 556 556 278 556 556 222 0 500 222 833 556 556
556 0 333 500 278 556 500 722 500 500 500 0 0 0 0 0
0 0 0 0 0 0 556 556 0 0 0 0 0 0 0 0
0 0 0 0 0 0 556]
/Encoding/WinAnsiEncoding/Subtype/Type1>>
endobj
10 0 obj
<</Type/FontDescriptor/FontName/MMJRJM+Helvetica-Oblique/FontBBox[0 -23 599 709]/Flags 4
/Ascent 709
/CapHeight 709
/Descent -23
/ItalicAngle 0
/StemV 89
/MissingWidth 278
/CharSet(/nine)/FontFile3 16 0 R>>
endobj
12 0 obj
```

```
<</Type/FontDescriptor/FontName/SNDABN+Times-Roman/FontBBox[0 0 1000 1000]/Flags 5
/Ascent 0
/CapHeight 0
/Descent 0
/ItalicAngle 0
/StemV 0
/AvgWidth 250
/MaxWidth 250
/MissingWidth 250
/CharSet(/space)/FontFile3 18 0 R>>
endobj
8 0 obj
<</Type/FontDescriptor/FontName/BCAJUP+Helvetica/FontBBox[-5 -218 859 741]/Flags 4
/Ascent 741
/CapHeight 741
/Descent -218
/ItalicAngle 0
/StemV 128
/MissingWidth 278
/CharSet(/two/A/y/n/c/three/z/o/d/four/N/C/p/e/five/O/D/f/six/P/E/r/g/seven/F/s/h/eight/R/endash/t/i/nine/S
/H/dagger/u/T/I/daggerdbl/v/k/semicolon/U/w/l/a/V/K/x/quotesingle/m/b/parenleft/parenright/asterisk/space/c
omma/hyphen/period/zero/percent/one)/FontFile3 20 0 R>>
endobj
2 0 obj
<</Producer(GPL Ghostscript 8.15)
/CreationDate(D:20081204100458)
/ModDate(D:20081204100458)
/Title(Microsoft Word - Document1)
/Creator(PScript5.dll Version 5.2.2)
/Author(heu5)>>endobj
xref
0 22
0000000000 65535 f
0000004174 00000 n
0000011250 00000 n
0000004105 00000 n
0000003944 00000 n
0000000015 00000 n
0000003924 00000 n
0000004222 00000 n
0000010797 00000 n
0000009818 00000 n
```

0000010335 00000 n
0000009492 00000 n
0000010558 00000 n
0000009658 00000 n
0000004263 00000 n
0000004293 00000 n
0000004345 00000 n
0000004833 00000 n
0000004853 00000 n
0000005225 00000 n
0000005245 00000 n
0000009471 00000 n
trailer
<< /Size 22 /Root 1 0 R /Info 2 0 R
/ID [(ç›T²ÀòVßÏç□ˆ…€u)(ç›T²ÀòVßÏç□ˆ…€u)]
>>
startxref
11445
%%EOF
